# KEY PREDICTORS OF COGNITIVE AGING

**DOI:** 10.1093/geroni/igac059.341

**Published:** 2022-12-20

**Authors:** Nicholas Turiano, Julie Blaskewicz Boron

## Abstract

Understanding who will experience cognitive dysfunction and dementia is as important as identifying methods to combat such deterioration. This symposium seeks to highlight new advances in understanding cognitive deterioration and promote resilience in adulthood. Odd and colleagues utilized a national sample (2,643 adults aged 34-85) to show that 9-year decreases in executive function and episodic memory predicted increased risk of dying. Use of a brief cognitive assessment administered via telephone was unique – a method that may assist clinicians administering cognitive screenings to older adults in isolated areas. Graham and colleagues utilized a daily diary approach in 116 older adults (aged 60-90) to show that greater daily fluctuations in mindfulness were associated with higher episodic memory and executive functioning. Further, mediational evidence suggested that on days when mindfulness was greater, individuals perceived a younger subjective age. Willroth and colleagues provide evidence that higher scores on eudaimonic well-being in older adults (n=349) predicted greater cognitive resilience. Specifically, even though some participants had neuropathological burden (e.g., increased beta-amyloid, neurofibrillary tangles), they did not exhibit pronounced cognitive declines. Using data from 14 longitudinal studies, Yoneda and colleagues examined the impact of physical activity on cognitive impairment and death. Multi-state survival analyses demonstrated engagement in more physical activity reduced risk of cognitive impairment and death. This symposium suggests that examining changes in cognition, incorporating subjective and objective indices of cognitive impairment, utilizing long-term longitudinal and daily diary designs, and testing key modifiable behaviors is crucial to understanding and promoting optimal cognitive functioning in adulthood.

